# The glutamine transporter ASCT2 (SLC1A5) promotes tumor growth independently of the amino acid transporter LAT1 (SLC7A5)

**DOI:** 10.1074/jbc.RA117.001342

**Published:** 2018-01-11

**Authors:** Yann Cormerais, Pierre André Massard, Milica Vucetic, Sandy Giuliano, Eric Tambutté, Jerome Durivault, Valérie Vial, Hitoshi Endou, Michael F. Wempe, Scott K. Parks, Jacques Pouyssegur

**Affiliations:** From the ‡ Medical Biology Department, Centre Scientifique de Monaco (CSM), MC 98000 Monaco,; § J-Pharma, Co. Ltd., Yokohama 230-0046, Japan,; ¶ School of Pharmacy, Anschutz Medical Campus, University of Colorado Denver, Aurora, Colorado 80045,; ‖ Institute for Research on Cancer and Aging (IRCAN), CNRS, INSERM, Centre A. Lacassagne, University of Nice Sophia Antipolis, 06088 Nice, France

**Keywords:** amino acid transport, cancer, mammalian target of rapamycin (mTOR), amino acid, cell metabolism, ASCT2, LAT1

## Abstract

The transporters for glutamine and essential amino acids, ASCT2 (solute carrier family 1 member 5, SLC1A5) and LAT1 (solute carrier family 7 member 5, SLC7A5), respectively, are overexpressed in aggressive cancers and have been identified as cancer-promoting targets. Moreover, previous work has suggested that glutamine influx via ASCT2 triggers essential amino acids entry *via* the LAT1 exchanger, thus activating mechanistic target of rapamycin complex 1 (mTORC1) and stimulating growth. Here, to further investigate whether these two transporters are functionally coupled, we compared the respective knockout (KO) of either LAT1 or ASCT2 in colon (LS174T) and lung (A549) adenocarcinoma cell lines. Although *ASCT2*^KO^ significantly reduced glutamine import (>60% reduction), no impact on leucine uptake was observed in both cell lines. Although an *in vitro* growth-reduction phenotype was observed in A549-*ASCT2*^KO^ cells only, we found that genetic disruption of ASCT2 strongly decreased tumor growth in both cell lines. However, in sharp contrast to *LAT1*^KO^ cells, *ASCT2*^KO^ cells displayed no amino acid (AA) stress response (GCN2/EIF2a/ATF4) or altered mTORC1 activity (S6K1/S6). We therefore conclude that *ASCT2*^KO^ reduces tumor growth by limiting AA import, but that this effect is independent of LAT1 activity. These data were further supported by *in vitro* cell proliferation experiments performed in the absence of glutamine. Together these results confirm and extend ASCT2's pro-tumoral role and indicate that the proposed functional coupling model of ASCT2 and LAT1 is not universal across different cancer types.

## Introduction

Commonly known as the building blocks of proteins, amino acids (AAs)^5^ are essential for biomass production because they also act as fundamental metabolites in the biosynthesis of lipids, nucleotides, glutathione, glucosamine, and polyamines, and also anaplerosis of the tricarboxylic acid (TCA) cycle (1, 2). Moreover, AAs (in particular leucine, arginine, and glutamine) act as signaling molecules because sufficient threshold levels are required to turn on the master regulator of cell metabolism and growth, mechanistic target of rapamycin complex 1 (mTORC1) (3–7). Therefore, cancer cells display an increased demand for AAs to produce the adequate biomass required to sustain their rapid proliferation. Importantly, the requirement for metabolite acquisition is even more exacerbated in the hypoxic tumor microenvironment because the availability of essential nutrients is further limited by the lack of regular blood perfusion (8). This constant selection pressure prompts tumor cells to evolve an increased ability to extract plasma-available AAs. This adaptation, coordinated both by oncogenic mutations (9–11) and stress response pathways (8, 12), leads to the overexpression of different AA transporters, an event which is now recognized as a hallmark of cancer metabolism (10).

Among the 30 AA transporters identified in human physiology, the L-type AA transporter 1 (LAT1/SLC7A5) and the alanine-serine-cysteine transporter 2 (ASCT2/SLC1A5) have emerged as major pro-tumoral transporters with increased expression levels correlating with poor patient prognosis in a large number of cancer types (13–16). ASCT2 is an Na^+^-dependent transporter that exchanges small neutral AAs (Ala, Ser, Cys, Gln, and Asn) (17). ASCT2 has been proposed to play a central role in sustaining glutamine metabolism and tumor growth (16, 18). Indeed, ASCT2 knockdown has been reported to inhibit mTORC1 activity and tumor growth in multiple xenograft models demonstrating the importance of this AA transporter during carcinogenesis (18, 20, 21). LAT1 is responsible for the Na^+^-independent uptake of essential AAs (Leu, Val, Ile, Phe, Trp, His, Met, Tyr) (22). LAT1 forms a heterodimer with the CD98 glycoprotein which acts as a chaperone promoting stabilization, trafficking, and functional insertion of LAT1 into the plasma membrane (22, 23). Work from our lab and others has demonstrated that LAT1 is essential for cancer cell proliferation by promoting AA homeostasis and mTORC1 activity (24, 25). Importantly, pharmacological inhibition or knockout of LAT1 strongly reduced tumor growth in several cancer cell types (24, 26). Interestingly, these two transporters are obligatory exchangers, meaning that the uptake of one AA is coupled with the efflux of another AA (17, 27). These exchange mechanisms quickly balance the cytoplasmic pools of AAs without expending energy and therefore give a strong advantage for cancer cell proliferation (28).

In 2009, Nicklin and colleagues (29) proposed a functional coupling of ASCT2 and LAT1 and demonstrated that cellular uptake of glutamine acts as a rate-limiting step in the essential AA-dependent activation of mTORC1. Indeed, ASCT2 was shown in HeLa cells to regulate an increase in the intracellular concentration of glutamine, which is secondarily used as an efflux substrate by LAT1 to promote the uptake of extracellular leucine and therefore the activation of mTORC1. This appealing concept of a functional coupling was reinforced by the fact that these two AA transporters are both c-Myc targets (30, 31). The functional coupling of ASCT2 and LAT1 is now cited extensively in the current literature (32–34). As this conclusion was derived from a single study and a unique cell line (HeLa) (29), we decided to further investigate this ASCT2/LAT1 functional coupling model. Recently, SNAT1/2 (SLC38A1/2) have been implicated as important players in maintenance of cellular glutamine levels in the absence of ASCT2, thus broadening the role of glutamine transport in cancer cells and mTORC1 activity (36).

Here we report that knockout of ASCT2 in two independent cancer cell lines (LS174T and A549) does not alter LAT1 transport activity. Moreover, whereas LAT1 is essential for cell proliferation by promoting AA homeostasis and mTORC1 activity, ASCT2 is dispensable *in vitro*. Interestingly, *ASCT2* ablation reduced tumor growth *in vivo* but this effect does not appear to be linked to a reduced LAT1 activity because no AA stress response and only a minor reduction of mTORC1 activity were detected in tumor tissue analyses. Together these findings demonstrate that the proposed functional coupling of ASCT2 and LAT1 is not obligatory nor a generalized phenomenon across cancer types. However, ASCT2 is required for optimal tumor growth *in vivo* and its ablation sensitized A549 cells to the LAT1 inhibitor JPH203 (24, 35), indicating that ASCT2 remains a promising target for cancer therapy.

## Results

### Genetic disruption of ASCT2 strongly reduces glutamine transport rates but does not alter LAT1 expression and activity

**Figure 1.**
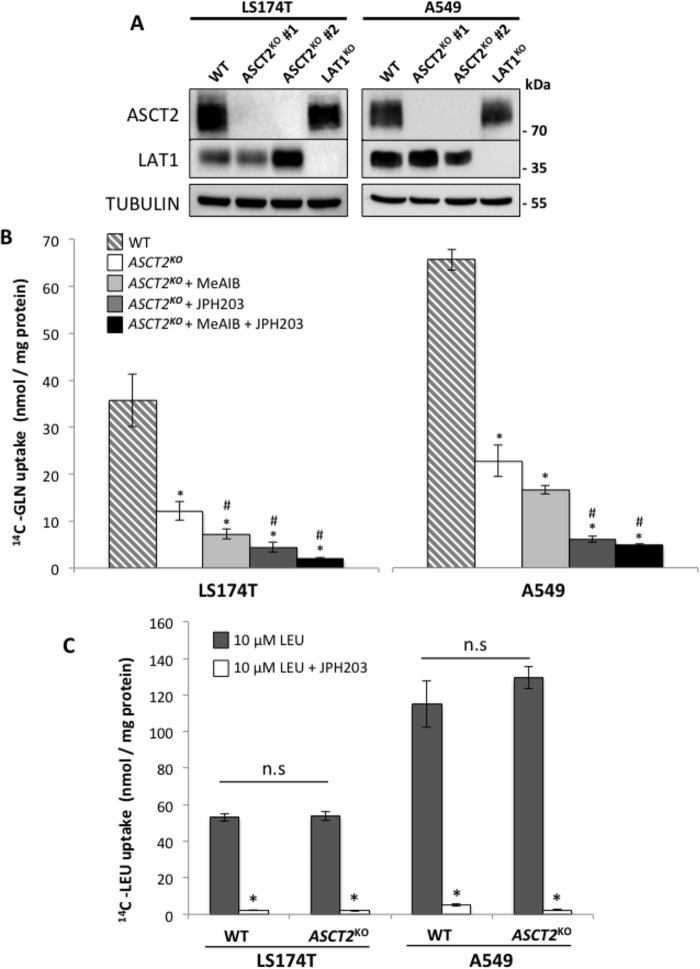
**Knockout of ASCT2 strongly decreases glutamine transport rate but does not alter LAT1 expression and activity.** *A*, the LAT1 and ASCT2 protein expression levels were analyzed by immunoblotting in LS174T and A549 WT, ASCT2^KO^ and *LAT1*^KO^ cells demonstrating an independence of protein expression between ASCT2 and LAT1. Two independent clonal cell lines of ASCT2^KO^ are shown (#1 and #2). Tubulin was used as a loading control. *B*, glutamine transport rates for WT and *ASCT2*^KO#1^ cells were measured by [^14^C]-glutamine uptake in HBSS media containing 10 μm glutamine. Inhibitors of system A (MeAIB) (10 mm) or LAT1 (JPH203) (30 μm) were used to identify transporters responsible for the residual glutamine transport activity in ASCT2 KO cells. *C*, LAT1 transport activity of WT and *ASCT2*^KO#1^ cells was measured by [^14^C]-leucine uptake in Na^+^-free HBSS media containing 10 μm leucine with (*white*) or without (*dark gray*) the LAT1 inhibitor JPH203 (30 μm). All of the above results represent the average of at least three independent experiments. *, significant compared with WT (*p* < 0.05); #, significant compared with untreated *ASCT2*^KO^ (ANOVA, *p* < 0.05); n.s., not significant.

*ASCT2* knockout (KO) was achieved in colon (LS174T) and lung (A549) adenocarcinoma cell lines using the CRISPR-Cas9 technique. To minimize clonal heterogeneity, experiments were performed on two independent clones. *LAT1*^KO^ cells were obtained from a previous study (24). Lack of corresponding protein expression (Fig. 1*A*), genomic DNA analysis, and sequencing of the CRISPR-targeted site demonstrated disruptive mutations in the *ASCT2* gene (Table S1). In both cell lines, removal of either ASCT2 or LAT1 does not appear to consistently influence the expression of their proposed functional partner (Fig. 1*A*). The impact of ASCT2 disruption on glutamine transport activity was then assessed (Fig. 1*B*). *ASCT2*^KO^ in both cell lines (*white bars*) reduced glutamine transport activity to ∼35% of WT cells (*dashed bars*), demonstrating that ASCT2 is the major glutamine transporter expressed in LS174T and A549 cells (Fig. 1*B*). Two different pharmacological inhibitors were then used to identify the transporters responsible for the residual glutamine transport activity in *ASCT2*^KO^ cells. These included JPH203, a specific LAT1 inhibitor (24, 35), and the AA analogue *N*-methylaminoisobutyric acid (MeAIB), an inhibitor of the system “A” AA transporters (SNAT1/SLC38A1, SNAT2/SLC38A2, and SNAT4/SLC38A5) (36). SNAT transporters mediate the Na^+^-dependent uptake of a wide variety of small neutral AAs and have been recently proposed as essential transporters for net glutamine uptake and proliferation of the osteocarcinoma cell line 143B (36). Utilization of these two inhibitors significantly reduced glutamine uptake in *ASCT2*^KO^ cells (*light gray*, *dark gray,* and *black bars*) demonstrating that LAT1 and SNATs contribute to the residual transport activity detected in *ASCT2*^KO^ cells (Fig. 1*B*).

To investigate the functional coupling of these transporters, the impact of the genetic disruption of ASCT2 on LAT1 activity was analyzed by measuring the Na^+^-independent rate of leucine transport (Fig. 1*C*). Surprisingly, despite the fact that glutamine uptake is strongly reduced, *ASCT2*^KO^ cells display the same leucine transport rates as WT cells (Fig. 1*C*). Combined, these results suggest that, even if its abolition strongly reduces glutamine uptake, ASCT2 is not required for LAT1 expression and activity in both LS174T and A549 cells.

### ASCT2 is not required for LAT1-dependent amino acid homeostasis, mTORC1 activity, and proliferation

**Figure 2.**
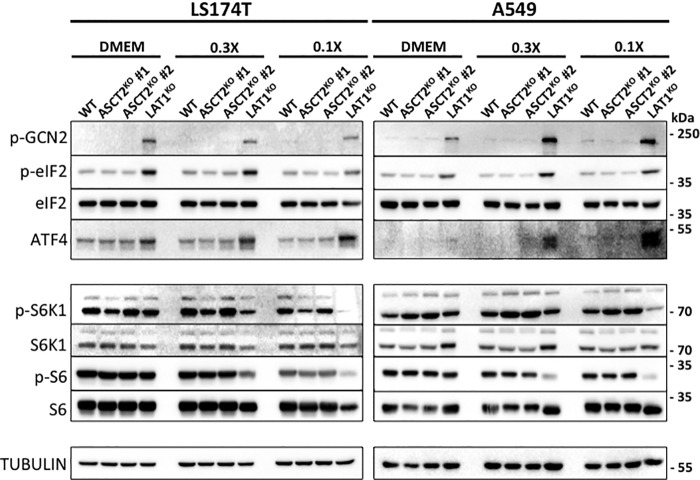
**LAT1 but not ASCT2 is required for amino acid homeostasis and mTORC1 activity *in vitro*.** WT, *ASCT2*^KO^, *LAT1*^KO^ cells were cultivated for 24 h in DMEM, 0.3× or 0.1× media (see “Experimental Procedures” for detailed composition). Changes in phosphorylation status and protein abundance of members of the two major amino acid sensing pathways GCN2 (p-GCN2/p-EIF2a/ATF4) and mTORC1 (p-S6K1 and p-S6) were analyzed by Western blotting. Tubulin was used as a loading control. Differential responses between cell lines are observed between culture media composition. The data presented are representative of a minimum of three independent experiments.

Previous analysis of *LAT1*^KO^ cells demonstrated that genetic disruption of LAT1 induces an AA stress response (GCN2/EIF2a/ATF4) and decreases mTORC1 activity (24). Because ASCT2 requirement for LAT1 activity has been reported (28, 29), we compared these AA sensing pathways in WT, *ASCT2*^KO^, and *LAT1*^KO^ cells (Fig. 2). In nutrient-rich media (DMEM), no AA stress response (observed through the phosphorylation of GCN2, EIF2a, and the expression level of ATF4) was detected in *ASCT2*^KO^ cell lines although this pathway is induced in *LAT1*^KO^ cells (Fig. 2). In addition, no significant changes in mTORC1 activity were observed (via the phosphorylation of S6K1 and S6) in all *ASCT2*^KO^ cells tested (Fig. 2). Next, cells were challenged with media in which the concentrations of AAs were closer to physiological levels (0.3× and 0.1×; see Table S2). In these two conditions, a strong induction of the GCN2 pathway and decreased mTORC1 activity was detected in *LAT1*^KO^ cells (Fig. 2). Again, induction of the GCN2 AA sensing pathway was not observed in *ASCT2*^KO^ cells. Furthermore, mTORC1 activity was not consistently modified except in A549-*ASCT2*^KO^ cells where the phosphorylation of S6K1 was slightly increased but its target S6 remained unchanged (Fig. 2). Finally, the activation kinetics of mTORC1 (measured via S6 phosphorylation) in response to glutamine preload and leucine uptake were compared in LS174T-WT, *ASCT2*^KO^, and *LAT1*^KO^ (Fig. S1A). As expected, *LAT1*^KO^ cells display a slower activation of mTORC1 whereas no significant differences were detected in *ASCT2*^KO^ cells, further confirming the data mentioned above. Furthermore, experiments performed with re-addition of glutamine alone following 24 h of glutamine starvation (see extended description below) revealed the same timing of mTORC1 activation between LS174T-WT and *ASCT2*^KO^ cells. Overall, these results demonstrate that the genetic disruption of *ASCT2* does not mimic the effect of *LAT1* knockout in terms of AA homeostasis and mTORC1 activity.

**Figure 3.**
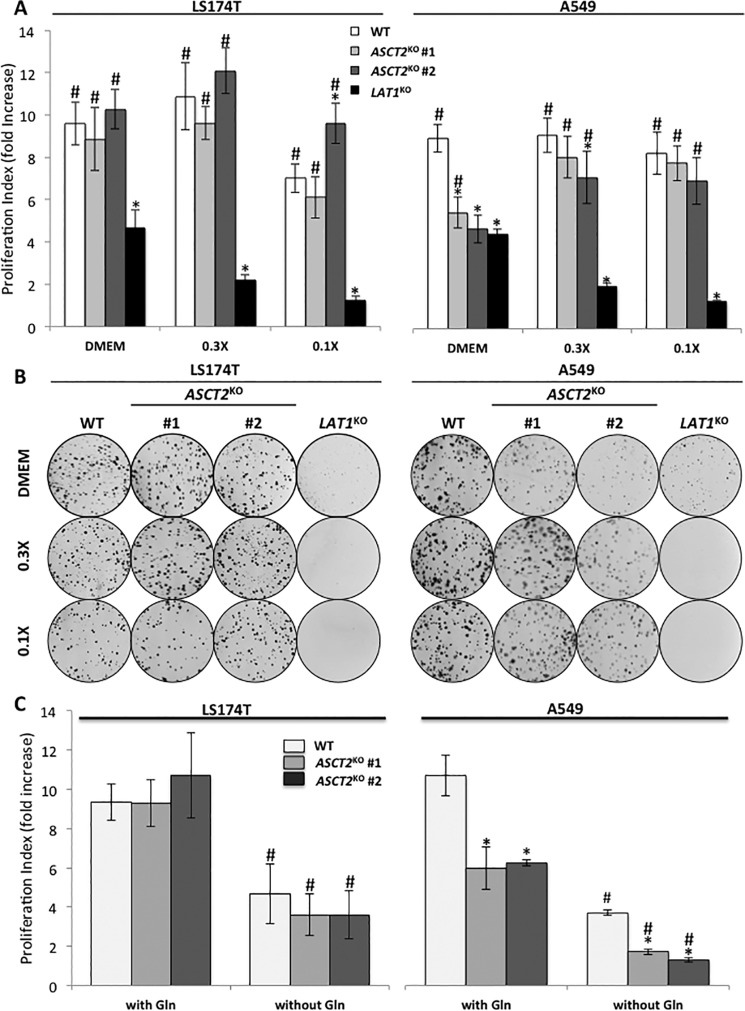
**ASCT2 knock-out does not phenocopy LAT1 knock-out *in vitro*.** *A*, cell proliferation of WT (*white*), *ASCT2*^KO^ (*light* and *dark gray*, two independent clones), and *LAT1*^KO^ (*black*) cells of LS174T and A549 cell lines. Cells were cultivated for 72 h in DMEM, 0.3×, or 0.1× media (Table S2). The media were replaced every day to maintain constant AA concentrations. Proliferation rates are presented as -fold increase (see “Experimental Procedures” for detailed description). These data represent the average of at least three independent experiments. *, significant compared with WT (ANOVA, *p* < 0.05), #, significant compared with *LAT1*^KO^ (*p* < 0.05). *B*, clonal growth of WT, *ASCT2*^KO^ (#1 and #2), and *LAT1*^KO^ cells of LS174T and A549 cell lines. Cells were cultivated for 15 days in DMEM, 0.3× or 0.1× media (Table S1). The media were replaced every 2 days to maintain constant AA concentrations and colored for visualization using Giemsa. *C*, cell proliferation of LS174T and A549 WT (*white*) and *ASCT2*^KO^ (*light* and *dark gray*, two independent clones) cells cultivated for 72 h in glutamine-free DMEM. These data represent the average of at least three independent experiments. *, significant compared with WT (ANOVA, *p* < 0.05); #, significant compared with DMEM (+) glutamine (*p* < 0.05).

We then compared the proliferation phenotype (-fold increase at 72 h) of WT (*white bars*), *ASCT2*^KO^ (*light* and *dark gray bars*), and *LAT1*^KO^ (*black bars*) cells (Fig. 3*A*). LS174T-*ASCT2*^KO^ cells displayed the same proliferation rate as WT cells in the three different media tested whereas *LAT1*^KO^ cell proliferation is strongly reduced (below 20% of WT rate in 0.3× and 0.1× media) (Fig. 3*A*). Interestingly, A549-*ASCT2*^KO^ cells exhibit the same reduced proliferation rate as *LAT1*^KO^ cells in standard DMEM (∼50%) (Fig. 3*A*). However, in AA reduced media (0.3× and 0.1×) A549-*ASCT2*^KO^ cells exhibited higher rates of proliferation matching the rate of WT cells whereas A549-*LAT1*^KO^ cell proliferation was below 20% the WT rate in 0.3× and 0.1× media) (Fig. 3*A*). Cell proliferation rates were further confirmed using 15-day clonogenecity assays (Fig. 3*B*). Together these findings demonstrate that genetic invalidation of *ASCT2* does not phenocopy the *LAT1*^KO^ cells *in vitro*, suggesting that ASCT2 is dispensable for LAT1-dependent AA homeostasis, mTORC1 activity, and proliferation.

To investigate the contribution of internal glutamine synthesis we monitored proliferation in the absence of external glutamine in standard DMEM. Glutamine removal reduced WT cell proliferation by ∼50% in both LS174 and A549 (Fig. 3*C*). LS174T-*ASCT2*^KO^ and WT cell proliferation rates were again the same in the absence of external glutamine whereas A549-*ASCT2*^KO^ cells maintained a lower proliferation rate in comparison to WT cells. The effect of glutamine starvation on mTORC1 activity and AA stress response was also monitored. Removal of external glutamine removed phosphorylation of S6K1 after 6 h and induced phosphorylation of GCN2 by 24 h in both WT and *ASCT2*^KO^ cells (Fig. S2), indicating that cellular glutamine synthesis is not sufficient to maintain mTORC1 activity and is not induced as an adaptive mechanism in *ASCT2*^KO^ cells. Inhibition of glutamine synthetase with methionine sulfoximine (MSO) in LS174 cells decreased mTORC1 activity after 1 h and continued for 6 h (Fig. S3). *ASCT2*^KO^ cells demonstrated the same mTORC1 activity patterns as WT cells in response to MSO treatment. Therefore, we conclude that the intracellular glutamine pool and synthesis rate cannot explain the inability of *ASCT2*^KO^ cells to phenocopy the AA stress response of *LAT1*^KO^ cells.

### ASCT2 is required for optimal tumor growth

**Figure 4.**
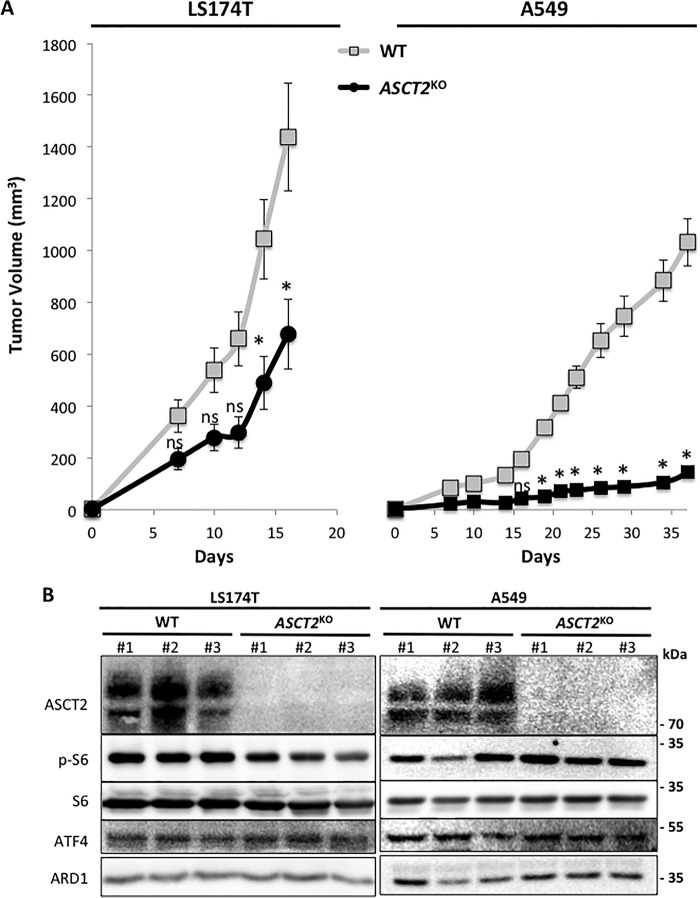
**ASCT2 is required for optimal tumor growth *in vivo*.** *A*, tumor volumes of nude mice injected subcutaneously with WT (*gray*) or *ASCT2*^KO^ (*black*) cells of the LS174T (*left*) and A549 (*right*) cell lines revealed an inhibition of tumor growth with ASCT2 knockout. *B*, protein levels of ASCT2, LAT1, and the two AA sensing pathways GCN2 (ATF4) and mTORC1 (p-S6) were analyzed by immunoblotting in three independent tumors from each LS174T and A549-derived cell line (WT and *ASCT2*^KO^). ARD1 acted as a protein loading control. *, significant compared with WT (ANOVA, *p* < 0.05).

To observe the impact of *ASCT2*^KO^ *in vivo* we utilized a mouse xenograft model. LS174T-WT, A549-WT and *ASCT2*^KO^ cells were injected subcutaneously into *nude* mice to monitor tumor growth (Fig. 4*A*). Surprisingly, although they display a normal proliferation rate *in vitro*, LS174T- and A549-*ASCT2*^KO^ tumors exhibited a reduced growth rate by ∼50 and 85%, respectively, compared with WT (Fig. 4*A*). To assess if this decrease in tumor size is linked to a reduced LAT1 activity, protein analysis was performed in three independent tumors of each cell type (Fig. 4*B*). LS174T-*ASCT2*^KO^ tumors exhibit a slightly reduced mTORC1 activity (observed via S6 phosphorylation) compared with the control tumors whereas the GCN2 pathway is not altered (observed via ATF4 levels). Remarkably, even with a near abolishment of growth (Fig. 4*A*), A549-*ASCT2*^KO^ tumors did not display any alterations in the AA sensing pathways mTORC1 and GCN2 (Fig. 4*B*). Previously we demonstrated that *LAT1*^KO^ tumor growth is strongly reduced (>90%) because of markedly decreased mTORC1 activity and activation of the GCN2 pathway (24). Therefore ASCT2 appears to be indeed required for optimal tumor growth of LS174T and A549 cells *in vivo*. However, this dependence does not appear to be linked to a modulation of LAT1 activity.

### Genetic disruption of ASCT2 sensitizes A549 cells but not LS174T to the LAT1 inhibitor JPH203

**Figure 5.**
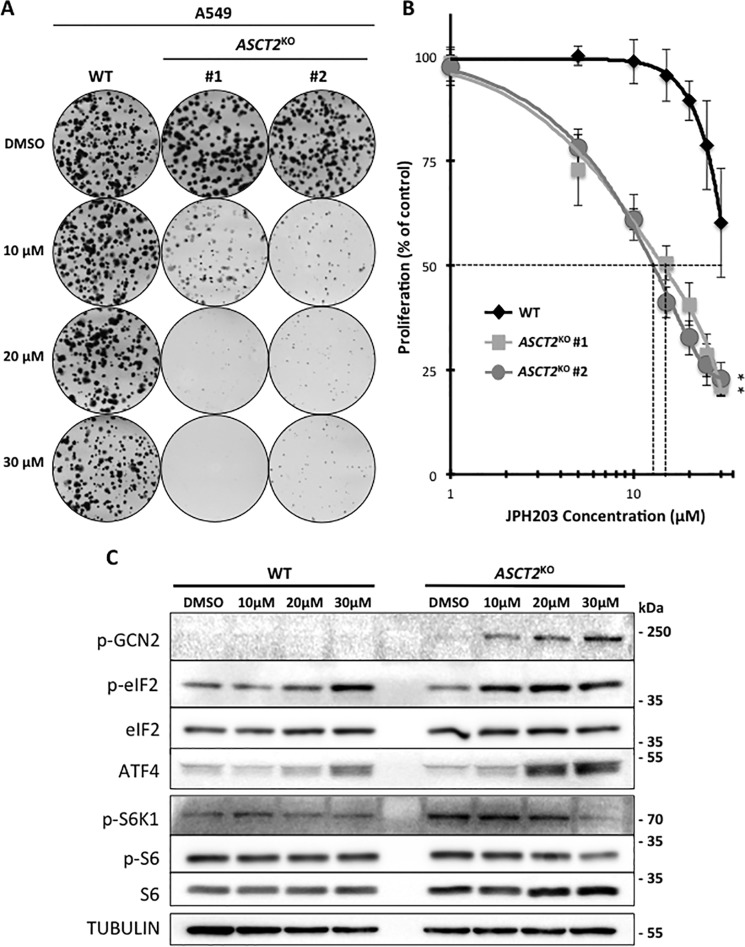
**A549-ASCT2^KO^ cells display an increased sensitivity to the LAT1 specific inhibitor: JPH203.** *A*, clonal growth of A549-WT and *ASCT2*^KO1 + 2^ cell lines. Cells were cultivated for 15 days with DMSO or different concentration of the LAT1-specific inhibitor (10, 20, 30 μm) in 0.3× media. The media were replaced every 2 days to maintain constant AA concentrations and colored for visualization using Giemsa. *B*, dose-response analysis of the LAT1-specific inhibitor JPH203 in A549-WT (*black*) and A549-*ASCT2*^KO^ (*gray*) cells. Cells were cultivated for 3 days in 0.3× DMEM containing different concentrations of inhibitor and cell numbers were counted to determine proliferation rates. At 5–30 μm JPH203, proliferation of the two *ASCT2*^KO^ clones was significantly decreased compared with WT cells (indicated by an asterisk). *C*, A549-WT and *ASCT2*^KO#1^ cells were cultivated for 48 h in 0.3× DMEM with DMSO or different concentrations of JPH203 (10, 20, 30 μm) for analysis of the amino acids sensing pathways GCN2 (p-GCN2/p-EIF2a/ATF4) and mTORC1 (p-p70-S6K1 and p-RPS6) via immunoblotting. Tubulin was used as a loading control.

As pharmacological inhibitors for LAT1 are progressing toward the clinic (11) we wanted to investigate the potential for a synergistic effect between LAT1 inhibition and ASCT2 disruption. Therefore, we analyzed the sensitivity of A549- and LS174T-*ASCT2*^KO^ cells to the specific LAT1 inhibitor JPH203 in 0.3× media. Interestingly, A549-*ASCT2*^KO^ cells display an increased sensitivity to JPH203 with an IC_50_ of ∼15 μm whereas the IC_50_ for A549-WT cells is greater than 30 μm (Fig. 5, *A* and *B*). To confirm this increased sensitivity in the context of AA stress we investigated the dose-response effect of JPH203 on the two AA sensing pathways investigated previously (GCN2 and mTORC1) in A549-WT and *ASCT2*^KO^ cells (Fig. 5*C*). Induction of the AA stress response pathway (GCN2) begins at 10 μm and is further increased at 30 μm in A549-*ASCT2*^KO^ cells whereas only a slight induction is detected in A549-WT cells at the highest dose. In terms of mTORC1 activity, 30 μm of JPH203 strongly decreased the phosphorylation of S6K1 and S6 in A549-*ASCT2*^KO^ cells whereas it has no effect on A549-WT. This increased sensitivity to LAT1 inhibition in *ASCT2*^KO^ cells appears to be cell line–specific because the sensitivity of LS174T-*ASCT2*^KO^ to JPH203 was not consistently altered (Fig. S2). Together these results demonstrate that ASCT2 disruption may sensitize certain tumor cell types to LAT1 inhibition suggesting that even if ASCT2 expression is not an obligatory requirement for LAT1-dependent AA homeostasis and proliferation, a certain level of functional coupling could exist. However, the difference between A549- and LS174T-*ASCT2*^KO^ cells in terms of JPH203 sensitivity suggests that other AA transporters or mechanisms may be involved in this functional AA transport collaboration.

## Discussion

Tumor cells that are “oncogenically rewired” for rapid growth continually sense overall nutrient availability. To survive, anabolic and catabolic processes are tightly controlled to efficiently coordinate growth with nutritional and energetic status. The key molecule within this coordination, often up-regulated in cancers, is mTORC1, a conserved serine-threonine kinase among eukaryotes (37, 38). mTORC1 integrates multiple inputs including growth factors, energy, oxygen, and nutrients (39). Although these exogenous factors have been extensively studied in the past, the influence of AAs such as leucine, arginine, and glutamine are currently the key research focal point of mTORC1 regulation. Thus, it is not surprising that a set of AA transporters has been implicated in regulation of mTORC1 activity and that their pharmacological inhibition has been proposed to reduce tumor progression.

Previously, our lab has demonstrated the essential role of LAT1 in promoting tumor growth via the maintenance of AA homeostasis and mTORC1 activity (24). Because LAT1 is an obligatory AA exchanger, its leucine uptake function relies on sufficient intracellular concentrations of the AA exchange substrates. Nicklin and colleagues (29) demonstrated that intracelluar glutamine is required to drive AA uptake via LAT1. Using HeLa cells, they proposed an interesting functional AA transporter-coupling model between ASCT2 and LAT1 whereby ASCT2 drives glutamine uptake, which in turn induces leucine import via LAT1, activating mTORC1 and growth. This model predicts that disruption of ASCT2 should inactivate LAT1. However, the knockouts of ASCT2 reported here in two cancer cell lines (LS174 and A549) (Fig. 2) do not satisfy this prediction. Importantly, *ASCT2*^KO^ was correlated with normal LAT1 leucine transport activity (Fig. 1*C*) and mTORC1 activity (Figs. 2 and S1) in both cell lines. Furthermore, whereas LS174 *LAT1*^KO^ cells exhibit a severe reduction in growth, *ASCT2*^KO^ cells are unaffected *in vitro* (Fig. 3, *A* and *B*). This absence of growth phenotype *in vitro* with *ASCT2*^KO^ was correlated with normal LAT1 activity (Fig. 1*C*) and mTORC1 activity (Figs. 2 and S1). A549-*ASCT2*^KO^ *in vitro* cell proliferation is indeed reduced in normal DMEM (Fig. 3, *A* and *B*) whereas mTORC1 activity was unaffected (Fig. 2). However, experiments in the absence of external glutamine (Figs. 3*C* and S2) show the same reduced growth rates for *ASCT2*^KO^ cells, indicating that the reduced growth phenotype is independent of glutamine transport as discussed further below. We also observed that in these glutamine-free growth conditions, *de novo* synthesis of glutamine enables low levels of cell proliferation; however there is no obvious compensation for up-regulated synthesis in *ASCT2*^KO^ cells as an adaptive pathway. We further confirmed that glutamine synthesis contributes to mTORC1 activity via use of the glutamine synthetase inhibitor MSO with no discernable differences between WT and *ASCT2*^KO^ cells (Fig. S3).

In line with our current findings, recent work on the osteocarcinoma cell line 143B-*ASCT2*^KO^ has also shown that ASCT2 is not required for mTORC1 activity (36). In this study, the absence of a growth phenotype was proposed to involve a functional redundancy of glutamine uptake via members of the SNAT transporting family. Therefore, glutamine cooperativity with LAT1 could potentially involve either the direct uptake via SNATs, ASCT2 transport, or a combination of the two. In our observations of glutamine uptake, we detected SNATs transporter activity in *ASCT2*^KO^ cells. Preliminary results from our lab show that only A549-*ASCT2*^KO^ cell growth is sensitive to the SNAT inhibitor MeAIB whereas only a slight effect is detected for LS174T cells (Fig. S5). These results suggest that A549-*ASCT2*^KO^ cells may rely on SNATs for glutamine uptake in the absence of ASCT2 as shown previously for 143B cells (36); LS174T cells may utilize yet another mechanism to drive LAT1 activity. Furthermore, additional glutamine transporters in A549 cells compared with LS174 could explain the elevated glutamine transport level in A549 cells (Fig. 1*B*). Our results combined with the literature suggest that the apparent absence of obligatory functional coupling between ASCT2 and LAT1 will require consideration of multiple glutamine transporters but also non-glutamine transporting mechanisms in future studies to obtain more precise information on mTORC1 activation. Our lab is currently exploring alternative glutamine transporter(s) or anabolic sources of AA capable to maintain import of leucine via LAT1. Consequently, it appears that variation between cancer types with respect to glutamine transport and potential cooperation with LAT1 may provide a formidable barrier to development of an effective, broad-reaching cancer-specific drug in this area. Nevertheless, ASCT2 inhibition could still prove to be useful as an additive therapy as A549-*ASCT2*^KO^ cells exhibited a notable increased sensitivity to LAT1 inhibition (Fig. 5, *A–C*). However, this increased sensitivity is not reproducible in LS174T-*ASCT2*^KO^ cells (Fig. S4, *A* and *B*), reinforcing the notion that other factor(s) can play a role in LS174 acquired resistance. Therefore effective LAT1 inhibition alone appears to be an area better suited for further research as it avoids the variable nature of transport cooperativity between cancer cell types and directly stops growth by inhibiting mTORC1 and the import of essential AAs (24), a feature common to all tumors.

Interestingly, despite an absence of a significant *in vitro* growth phenotype for *ASCT2*^KO^ cells, the *in vivo* tumor growth was strongly altered (Fig. 4*A*). This growth reduction does not correspond however to what was previously described for *LAT1*^KO^ tumors (24). Indeed, the two AA sensing pathways GCN2 and mTORC1 were not significantly modified in *ASCT2*^KO^ tumors (Fig. 4*B*), correlating with our *in vitro* results demonstrating that ASCT2 is dispensable for LAT1-dependent AA homeostasis and mTORC1 activity. These results highlight the metabolic differences between *in vitro* and *in vivo* conditions for cancer cells and suggest that *in vivo*, ASCT2 AA transport activity is required for tumor growth independently of LAT1. Historically, ASCT2 literature has focused on its role in glutamine uptake, leading to the assumption that ASCT2 disruption would alter glutamine metabolism and thus tumor growth (16, 18). However, this concept and our current data (especially in A549 tumors) oppose what has been recently found in a wide array of lung cancer cell studies where *in vitro* cell proliferation typically requires enhanced glutamine supply for the TCA cycle whereas *in vivo* growth relies primarily on glucose-derived pyruvate to fuel the TCA cycle (40–42). Therefore, the *in vivo* phenotype of *ASCT2*^KO^ cells may not reflect a decreased glutamine uptake but rather an alteration in other AA substrates. This is supported by our *in vitro* data showing reduced proliferation in *ASCT2*^KO^ cells even in the complete absence of external glutamine (Fig. 3*C*). Indeed, ASCT2 carries a number of other substrates and notably the two semi-essential AAs: serine and cysteine (17). Despite the fact that they can be *de novo* synthetized, exogenous serine and cysteine have been shown to play an important role in supporting viability and proliferation of certain cancer cells (43–46). Serine uptake is enhanced in cancer cells to be utilized as an intermediate metabolite for nucleotide synthesis (43, 44). Cysteine is the rate-limiting substrate for glutathione synthesis, the key nonenzymatic cellular defense molecule against oxidative stress (45, 46). Therefore, further investigation is required to measure the potential implication of ASCT2 in sustaining cysteine and serine metabolism *in vivo* and thereby tumor growth.

In summary, our study demonstrates that although ASCT2 activity is required for optimal tumor growth, thus rendering it a potentially good target for cancer therapy, the proposed functional coupling of ASCT2 and LAT1 is not obligatory across cancer types, a conclusion that extends the recent report of Broër *et al.* (36). This concept of functional collaboration between amino acid carriers likely exists but the large spectrum of substrates and redundant mechanisms results in an incredibly complex challenge in the design of an AA transporter model that is common for all tumor types. Therefore future efforts must focus on investigating multiple AA substrates and transporters in combination to uncover the true potential of targeting AA transporters for effective cancer therapy. Ultimately, it appears that disruption of AA transport mechanisms remains a promising target because of its essential role in tumor progression.

## Experimental procedures

### Cell culture

Colon adenocarcinoma LS174T cells (kindly provided by Dr. Van de Wetering, NL), and A549 cells (obtained from American Type Culture Collection, Manassas, VA) were used in all experiments. These two cell lines have been authenticated by DNA profiling of eight highly polymorphic short tandem repeat loci (DSMZ, Germany). Cells were grown in DMEM (Gibco) supplemented with 7.5% dialyzed FBS, penicillin (10 units/ml), and streptomycin (10 μg/ml) and MEM NEAA (Gibco). 0.3× media was obtained by mixing 2 volumes of DMEM lacking all amino acids with 1 volume of DMEM without glutamine. The 0.1× media was obtained by mixing 9 volumes of DMEM lacking all amino acids with 1 volume of DMEM without glutamine. Glutamine was added fresh just before cell culture experiments respectively at 4 mm (DMEM) 500 μm (0.3×) and 166 μm (0.1×). For the final concentration of AA in the different media used in this study and comparison with plasma concentrations see Table S2.

### Genomic disruption of ASCT2 using CRISPR-Cas9

LS174 and A549 WT cells were transfected with PX458 plasmids containing CRISPR-Cas9 targeting regions of the fifth exon of the ASCT2 (SLC1A5) gene using JetPRIME (Polyplus). The pSpCas9(BB)-2A-GFP (PX458) plasmid was a gift from Dr. Feng Zhang (Addgene plasmid no. 48138) (19). The sgRNA sequence that we cloned into the vector to target ASCT2 was 5′-GC GGC GTC ACG ATG CCC CAC-3′. As the PX458 plasmid contains GFP, cells were first immediately sorted using flow cytometry to obtain cells containing the CRISPR-Cas9. Cells were plated in clonal conditions (250 individualized cells in 100-mm dishes). Each clone was picked and analyzed for ASCT2 expression by immunoblotting and negative clones were re-cloned and further analyzed by DNA sequencing (Table S1). LAT1 knockout cells were obtained from our previous study (24).

### Glutamine starvation and inhibition of cellular glutamine synthesis

Cells were exposed to glutamine deprivation experiments in the following manner. For cell proliferation studies, the effect of glutamine-free DMEM on LS174 and A549 WT+*ASCT2*^KO^ cells was followed for 72 h as described below. Glutamine starvation (in otherwise complete DMEM supplemented with MEM NEAA) was also performed for analysis of the AA stress response and mTORC1 activation. Finally, the glutamine synthetase inhibitor MSO was used (3 mm) in complete DMEM supplemented with MEM NEAA for observation of mTORC1 activity during 0–6 h.

### Immunoblotting

Cells were lysed in 1.5× Laemmli buffer and protein concentrations were determined using the Pierce BCA protein assay (23227, Thermo Scientific). Protein extracts were separated by electrophoresis on 10% SDS-polyacrylamide gel and transferred onto polyvinylidene difluoride membranes (EMD Millipore). Membranes were blocked in 5% nonfat milk in PBS buffer (50 mm Tris-HCl pH7.4, 150 mm NaCl) and incubated with the following anti-human antibodies: rabbit LAT1 (1:1000, KE026, TransGenic Inc.), rabbit ASCT2 (1:1000, 8057S, Cell Signaling Technology (CST)), mouse GCN2 (1:250, sc-374609, Santa-Cruz Biotechnology), mouse phospho-GCN2 (1:500, ab75836, Abcam), rabbit EIF2α (1:1000, ab5369, Abcam), mouse phospho-EIF2α (1:1000, ab32157, Abcam), rabbit ATF4 (1:1000, 11815S, CST), rabbit p70-S6K1 (1:1000, 9202S, CST), rabbit phospho-p70-S6K1 (1:1000, 9204S, CST), rabbit S6 (1:1000, 2217S, CST), and rabbit phospho-S6 (1:1000, 2215S, CST). Detection of tubulin (1:10000, MA5–16308, Thermo Scientific) or ARD1 (47) was used as a protein loading control. Immunoreactive bands were detected with horseradish peroxidase (HRP) anti-mouse or anti-rabbit antibodies (CST) using the ECL system (Merck Millipore WBKLS0500). Analysis and quantification of immunoblotting were performed using the LI-COR Biosciences Odyssey Imaging System.

### l-[^14^C]-amino acid uptake

Cells (5 × 10^5^) were seeded onto 35-mm dishes, in triplicate per cell line and used for uptake experiments 24 h after seeding. Culture media were removed and cells were carefully washed with prewarmed Hanks' Balanced Salt Solution (HBSS) (137 mm NaCl, 1.3 mm CaCl_2_, 0.5 mm MgCl_2_, 0.4 mm MgSO_4_, 5.3 mm KCl, 0.44 mm KH_2_PO_4_, 4.2 mm NaHCO_3_, 0.33 mm Na_2_HPO_4_, 5.6 mm glucose, 25 mm HEPES), pre-incubated in 1.0 ml of prewarmed HBSS at 37 °C for 5 min before adding substrates for the uptake experiment. Cells were then incubated at 37 °C for 5 min in 1 ml of HBSS containing 10 μm L-[^14^C]-amino acid (0.03 μCie/ml) (PerkinElmer Life Sciences). Subsequently, cells were washed three times with ice-cold Na^+^-free HBSS containing 1.0 mm nonradiolabeled amino acid. Cells were then lysed with 500 μl of 0.1 N NaOH and mixed with 3 ml of Ultima Gold (PerkinElmer). Radioactivity was measured using a β-scintillation counter. Specifically, L-[^14^C]-leucine uptake was performed with a Na^+^-free HBSS media (125 mm choline chloride, 4.8 mm KCl, 1.2 mm MgSO_4_, 1.2 mm KH_2_PO_4_, 1.3 mm CaCl_2_, 5.6 mm glucose, 25 mm HEPES). Inhibition experiments were performed in the presence of 10 mm MeAIB and/or 30 μm JPH203.

### Proliferation assay

The different cell lines (2.5 × 10^4^ cells for A549 and 4 × 10^4^ for LS174) were seeded onto 6-well plates in triplicate per cell line and per condition. We measured proliferation by trypsinizing the cells and counting them daily with a Coulter Z1 (Beckman) during 3 days. The cell proliferation index was calculated as -fold increase by standardizing each measurement to the cell number obtained 24 h after seeding (Day 0).

### LAT1 inhibitor dose response assay

The different cell lines (2.5 × 10^4^ cells for A549 and 4 × 10^4^ for LS174) were seeded onto 6-well plates in triplicate for each cell line and JPH203 concentrations indicated. We measured proliferation as described above.

### Clonogenicity assay

LS174 and A549-derived mutants (1000 cells) were plated in 60-mm dishes and incubated at 37 °C, 5% CO_2_. 24 h after cell adherence, the media were replaced with specified media (Table S2) supplemented with 7.5% dialyzed serum and containing JPH203 for LAT1 inhibition experiments. Media were changed every 2 days. Dishes were stained with 5% Giemsa (Fluka) for 30–45 min to visualize colonies.

### Tumor xenograft studies

The different stable cancer cell lines (1 × 10^6^ cells) suspended in 500 μl of serum-free DMEM supplemented with insulin-transferrin-selenium (Life Technologies) were injected subcutaneously into the backs of 8-week-old female athymic mice (Janvier). Tumor dimensions were measured at least twice a week using calipers and the tumor volume was determined by using the formula: (4π/3) × L/2 × W/2 × H/2, (L, length; W, width; and H, height). When the tumor volume reached 1000 mm^3^, mice were euthanized and the tumors were excised. For protein analysis, tumors were lysed directly after harvesting. Tumors were incubated in cell extraction buffer (FNN0011, Thermo Scientific) supplemented with Halt protease inhibitor mixture (78429, Thermo Scientific) and lysed using a Precellys homogenizer. Animal housing was done in compliance with the European Union directive 2010/63/EU. Briefly, each cage contained five mice with an enriched environment, food and water were given *ad libitum*, and the litter was changed on a weekly basis. Animal care met the European Union directive 2010/63/EU ethical criteria. The animal experimentation protocol was approved by the local animal care committee. (Veterinary service and direction of sanitary and social action of Monaco Dr. H. Raps.)

### Statistical analysis

Data are expressed as mean ± S.D. Each experiment was performed at least three times. Statistical analysis was performed with either one or two-way analysis of variance (ANOVA) using the Bonferroni and Dunnett's post hoc tests where appropriate. Differences between groups were considered statistically significant when *p* <0.05.

## Author contributions

Y. C., P. A. M., M. V., S. K. P., and J. P. conceptualization; Y. C., P. A. M., M. V., and S. K. P. data curation; Y. C., P. A. M., M. V., S. G., S. K. P., and J. P. formal analysis; Y. C., P. A. M., M. V., S. G., E. T., J. D., V. V., and S. K. P. investigation; Y. C., M. V., S. K. P., and J. P. methodology; Y. C., S. K. P., and J. P. writing-original draft; Y. C., M. V., S. K. P., and J. P. writing-review and editing; H. E. and M. F. W. resources; S. K. P. and J. P. supervision; J. P. funding acquisition; J. P. project administration.

## Supplementary Material

Supporting Information

## Footnotes

5 The abbreviations used are:

AA: amino acid
TCA: tricarboxylic acid
mTORC1: mechanistic target of rapamycin complex 1
MeAIB: *N*-methylaminoisobutyric acid
MSO: methionine sulfoximine
sgRNA: single guide RNA
ANOVA: analysis of variance.
