# Supplementary material for: The glutamine transporter ASCT2 (SLC1A5) promotes tumor growth independently of the amino acid transporter LAT1 (SLC7A5)
Source: J Biol Chem. 2018 Jan 11;293(8):2877–87. doi: 10.1074/jbc.RA117.001342 (PMC5827425; doi:10.1074/jbc.RA117.001342)
Supplement: Supporting Information [file supp_293_8_2877__index.html]

The glutamine transporter ASCT2 (SLC1A5) promotes tumor growth independently of the amino acid transporter LAT1 (SLC7A5) — ASCT2 and LAT1 independantly control tumor growth — The glutamine transporter ASCT2 (SLC1A5) promotes tumor growth independently of the amino acid transporter LAT1 (SLC7A5) — ASCT2 and LAT1 independently control tumor growth — Supporting Information 

# The glutamine transporter ASCT2 (SLC1A5) promotes tumor growth independently of the amino acid transporter LAT1 (SLC7A5)

## Supporting Information

- Supplementary Legends - Supplementary Table and Figure legends
- Supporting Information merged
