# Supplementary material for: The glutamine transporter ASCT2 (SLC1A5) promotes tumor growth independently of the amino acid transporter LAT1 (SLC7A5)
Source: J Biol Chem. 2018 Jan 11;293(8):2877–87. doi: 10.1074/jbc.RA117.001342 (PMC5827425; doi:10.1074/jbc.RA117.001342)
Supplement: Supporting Information [file supp_RA117.001342_134499_1_supp_45063_p1m4dl.pdf]

Supplementary Tables & Figure legends

Table S1

Summary of the mutations introduced by CRISPR/Cas9 in each LS174T- and A549-*ASCT2*^{KO} clones used. *LAT1*^{KO} clones where characterized in a previous study (24)

Table S2

Amino acid concentrations for each of the culture media used in this study are described. These concentrations are compared with physiological concentration summarized in (48)

Figure S1

A: Time-dependent activation points of mTORC1 were compared between LS174T-WT -*ASCT2*$^{KO\#1}$ and *LAT1*^{KO} cells. Cells were first serum- and AA-starved for 2 hours followed by 20 min of pre-incubation with 500 µM of glutamine. LS174T-derived cells were then incubated with 200 µM of leucine for 0, 5,10 and 20 minutes and mTORC1 activity was measured through the phosphorylation level of S6.

B: Cells were starved of glutamine only in DMEM for 24h and then glutamine was re-introduced to observe the impact on mTORC1 activity over 10, 30, and 120 minutes as described for the LS174 cells types in part A. WB data presented are representative of 3 independent experiments.

Figure S2

Induction of the AA-stress response pathway and reduction of mTORC1 activity in LS174T cells (WT/*ASCT2*^{KO}/*LAT1*^{KO}) occurs in the absence of external glutamine. Cells were subjected to glutamine removal only in DMEM and the AA-stress response pathway (p-GCN2/eIF2/ATF4) and mTORC1 activity (p-S6K1/p-S6) were monitored for 6, 24 and 30 hours. HSP90 served as a loading control. Data presented are representative of 3 independent experiments.

Figure S3

The glutamine synthetase inhibitor methionine sulfoximine (MSO) decreases S6K1 phosphorylation in LS174 WT- *ASCT2*^{KO} and *LAT1*^{KO} cells. MSO (3mM) was applied to cells grown in DMEM containing 500µM and mTORC1 activity was observed for 1, 3 and 6h by monitoring S6K1/S6 phosphorylation with ARD1 acting as an additional loading control. Data presented are representative of 3 independent experiments.

Figure S4

A: Clonal growth of LS174T-WT and *ASCT2*^{KO} cell lines. Cells were cultivated for 15 days with DMSO or different concentration of the LAT1 specific inhibitor (10, 20, 30µM) in 0.3X media. The media was replaced every 2 days to maintain constant AA concentrations and colored for visualization using Giemsa.

B: Dose response analysis of the LAT1 specific inhibitor JPH203 in A549-WT (black) and A549-*ASCT2*^{KO} (grey) cells. Cells were cultivated for 3 days in 0.3X DMEM containing different concentrations of inhibitor and cell numbers were counted to determine proliferation rates. Data presented in this figure are combined from a minimum of 3 independent experiments.

Figure S5

Clonal growth of WT and *ASCT2*^{KO} cell lines. Cells were cultivated for 15 days with different concentration of the SNATs inhibitor MeAIB (1, 5, 10 mM) in 0.3X media. The media was replaced every 2 days to maintain constant AA concentrations and colored for visualization using Giemsa.
