# Supplementary material for: The glutamine transporter ASCT2 (SLC1A5) promotes tumor growth independently of the amino acid transporter LAT1 (SLC7A5)
Source: J Biol Chem. 2018 Jan 11;293(8):2877–87. doi: 10.1074/jbc.RA117.001342 (PMC5827425; doi:10.1074/jbc.RA117.001342)
Supplement: Supporting Information [file supp_RA117.001342_134499_1_supp_51641_p2wtv2.pdf]

	Plasma*	DMEM#	0.3X	0.1X
**Alanine**	146 - 494	100	100	33,3
**Arginine**	28-132	400	133,3	40
**Asparagine**	32-92	100	100	33,3
**Aspartate**	2-22.5	100	100	33,3
**Cystine**	24--58.3	200	66,7	20
**Glutamate**	6-62	100	100	33,3
**Glutamine**	450-798	4000	500	166,7
**Glycine**	147-299	400	133,3	40
**Histidine**	72-108	200	66,7	20
**Isoleucine**	46-122	800	266,7	80
**Leucine**	79-205	800	266,7	80
**Lysine**	135-243	800	266,7	80
**Methionine**	13-37	200	66,7	20
**Phenylalanine**	46-84,8	400	133,3	40
**Proline**	97-297	100	100	33,3
**Serine**	85-165	400 (+100)	133,3(+100)	40(+33,3)
**Threonine**	92-180	800	266,7	80
**Tryptophan**	21-65	80	26,7	8
**Tyrosine**	37-82,8	400	133,3	40
**Valine**	179-335	800	266,7	80

* : From J.R. Mayers and M.G. Vander Heiden, Trends Biochem Sci. 2015

# : DMEM supplemented with MEM NEAA
Concentration in µM

Table S1

**Clone**	**Allele**	**Mutation type**	**Mutation Sequence**
**LS174T-ASCT2^{KO} #1**	1	Deletion	GG
	2	Deletion	GTGGGGCA
**LS174T-ASCT2^{KO} #2**	1	Deletion	G
	2	Deletion	GG
**A549-ASCT2^{KO} #1**	1	Deletion	G
	2	Deletion	TGTG
**A549-ASCT2^{KO} #2**	1 & 2	Deletion	GG

Targeted sequence : ASCT2 exon 5 : GTGGGGCATCGTGACGCCGC

Table S2

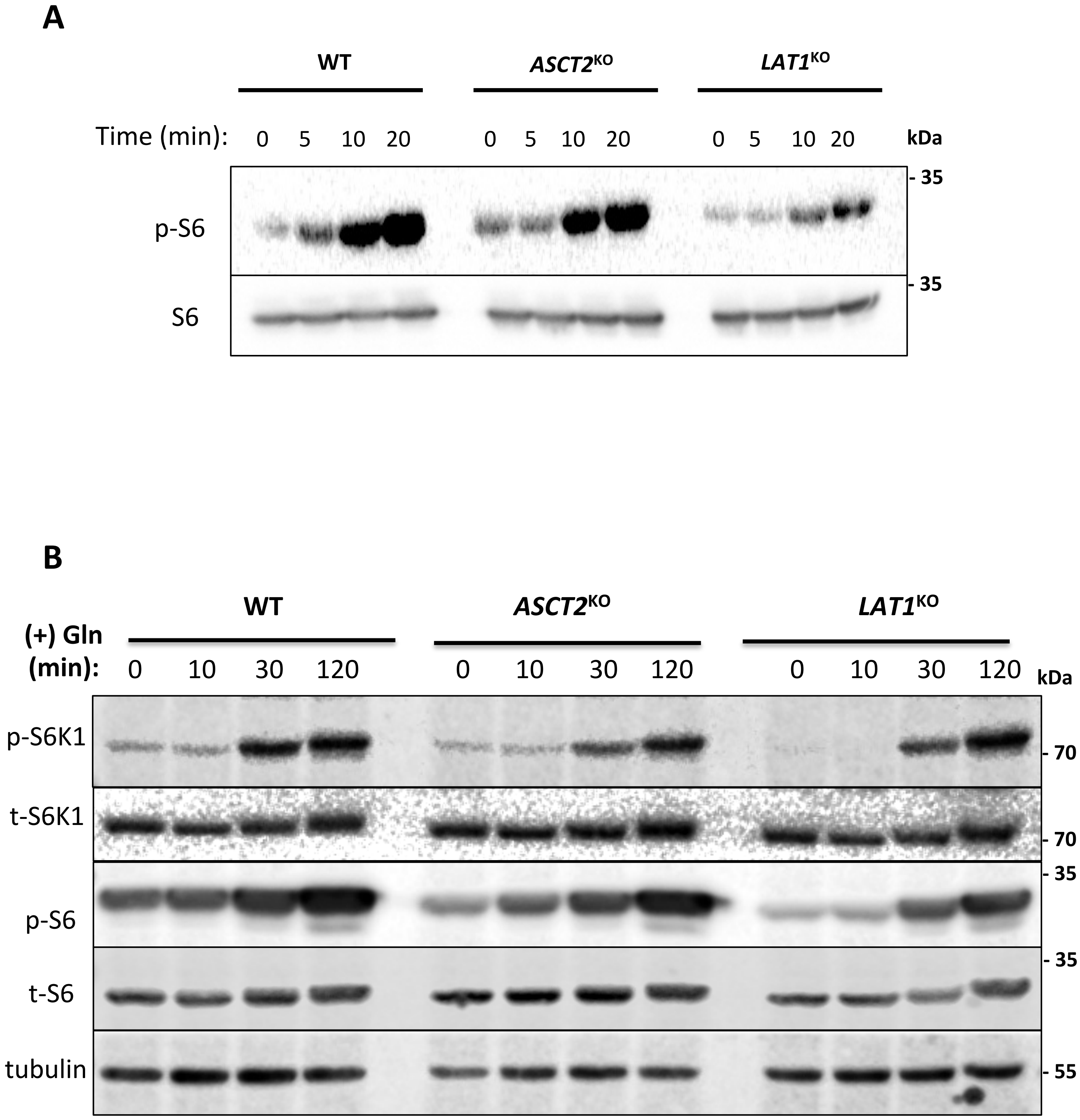
A
WT
ASCT2KO
LAT1KO
Time (min): 0 5 10 20 0 5 10 20 0 5 10 20
kDa
p-S6
S6
- 35
- 35
B
WT
ASCT2KO
LAT1KO
(+) Gln (min): 0 10 30 120 0 10 30 120 0 10 30 120
kDa
p-S6K1
t-S6K1
p-S6
t-S6
tubulin
- 70
- 70
- 35
- 35
- 55

Figure S1

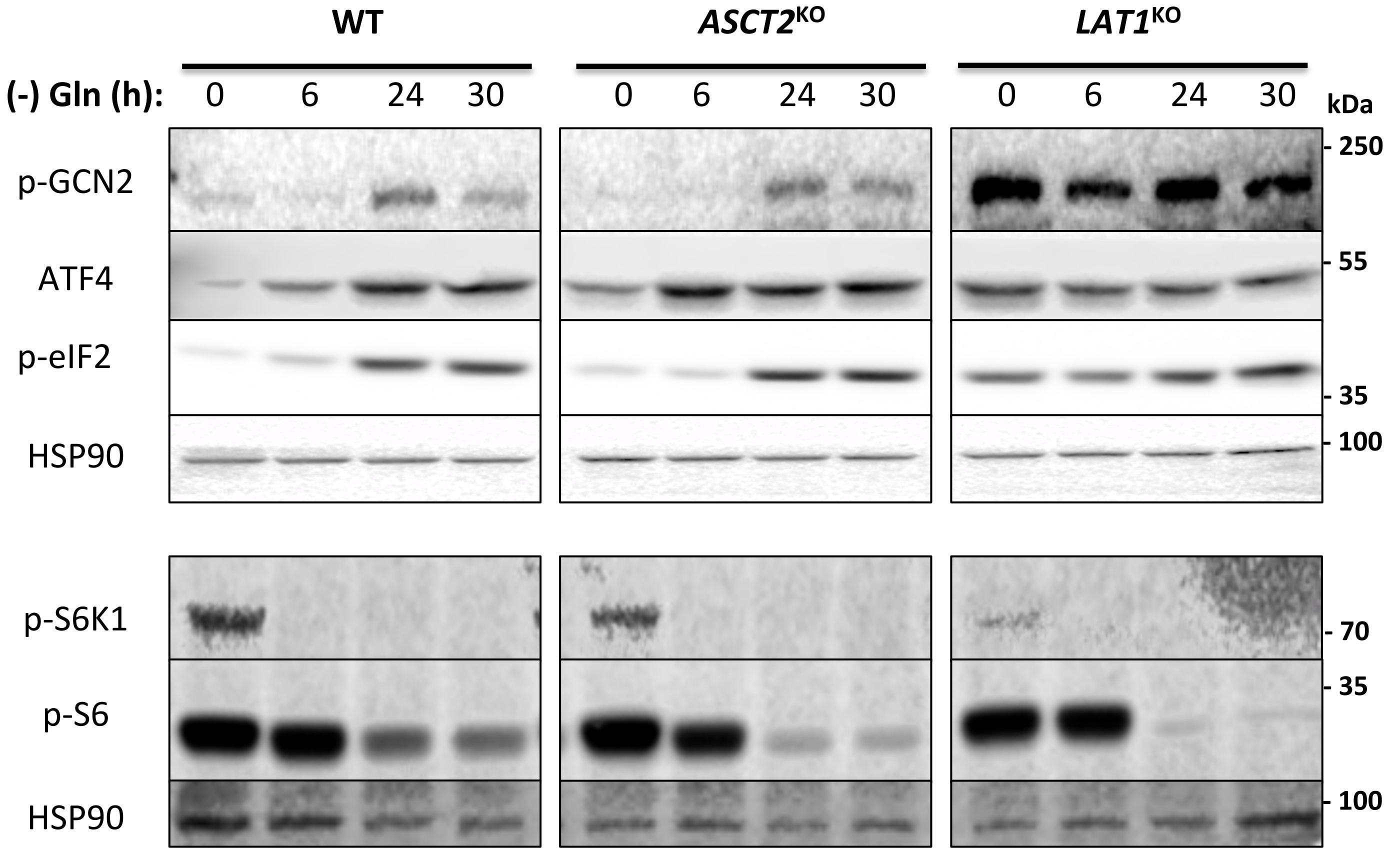
WT
*ASCT2*KO
*LAT1*KO
(-) Gln (h):
0
6
24
30
0
6
24
30
0
6
24
30
kDa
p-GCN2
- 250
ATF4
- 55
p-eIF2
- 35
HSP90
- 100
p-S6K1
- 70
p-S6
- 35
HSP90
- 100

Figure S2

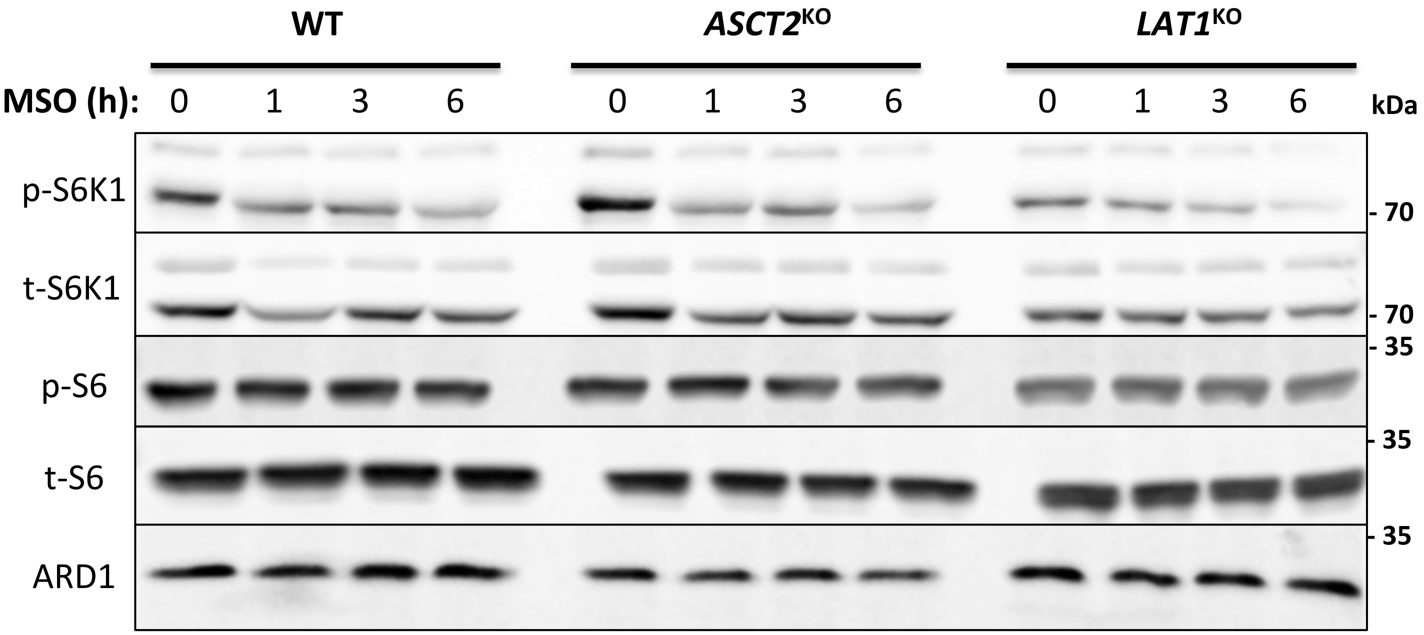
WT
ASCT2KO
LAT1KO
MSO (h):
0 1 3 6
0 1 3 6
0 1 3 6
kDa
p-S6K1
t-S6K1
p-S6
t-S6
ARD1
70
70
35
35
35

Figure S3

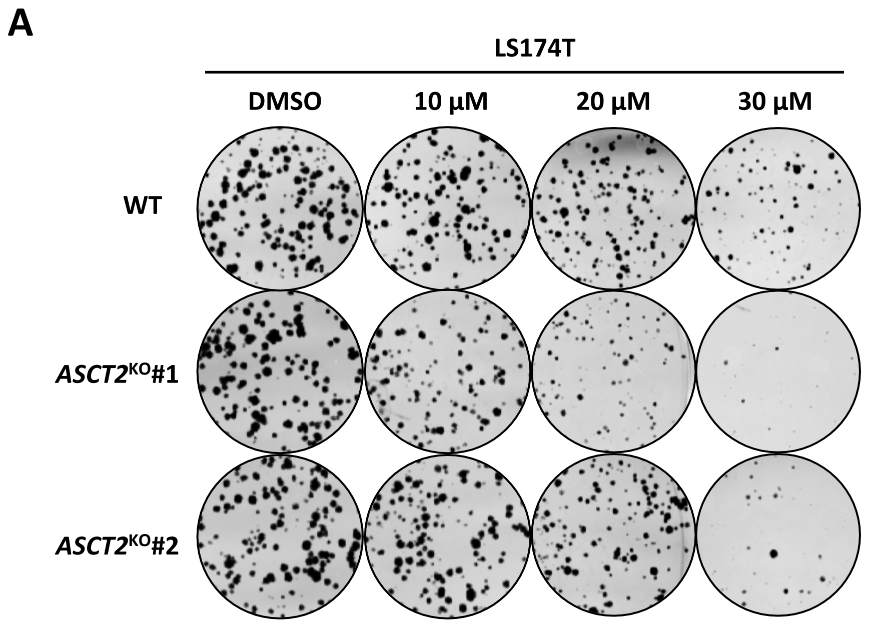
A
LS174T
DMSO
10 µM
20 µM
30 µM
WT
ASCT2KO#1
ASCT2KO#2

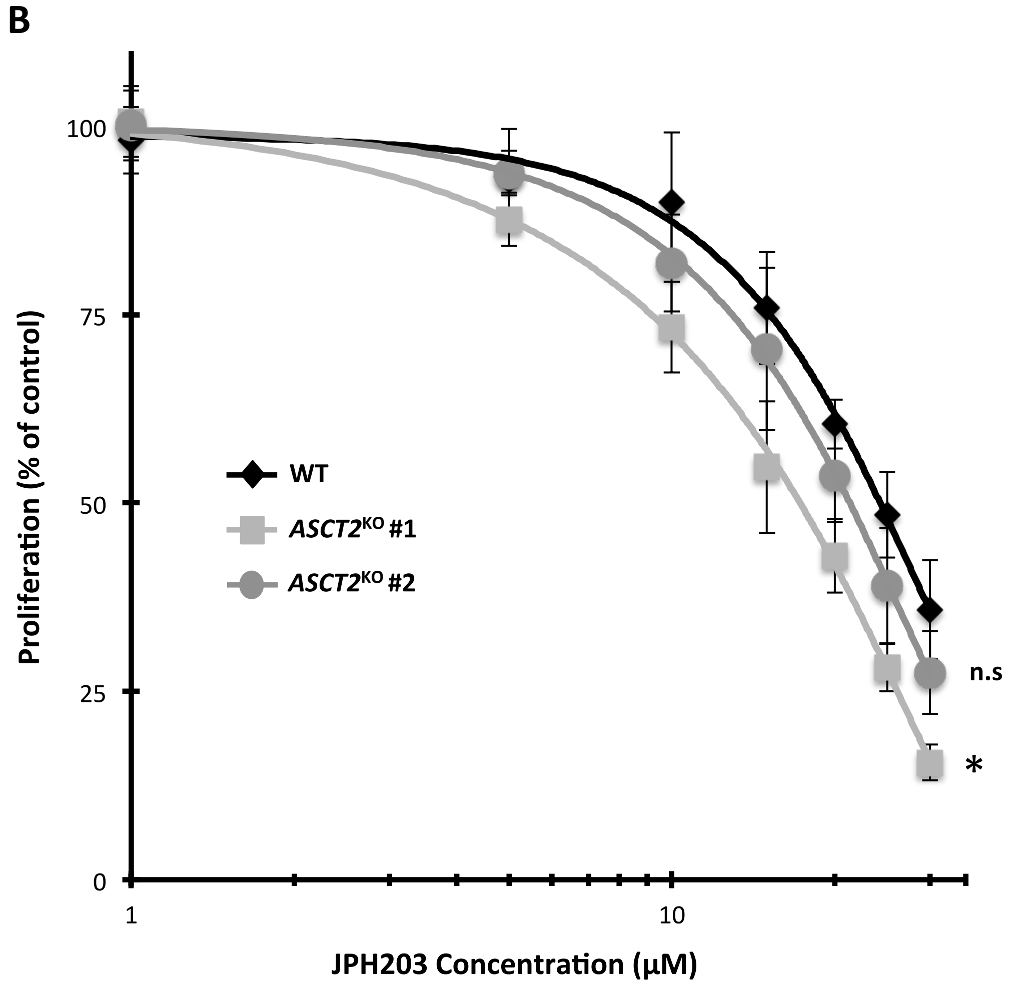
B
Proliferation (% of control)
100
75
50
25
0
1
10
JPH203 Concentration (µM)
WT
ASCT2KO #1
ASCT2KO #2
n.s
*

Figure S4

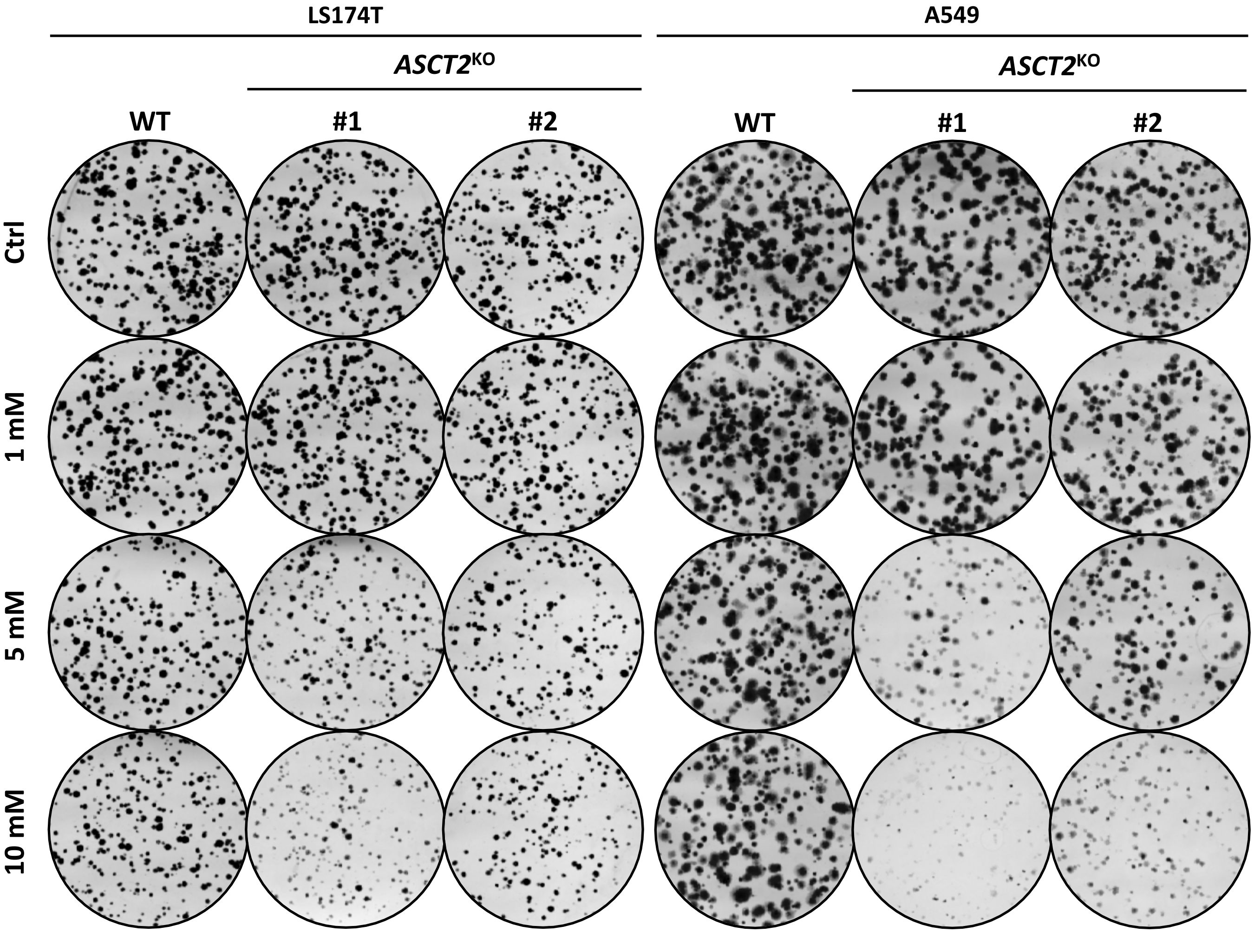

Figure S5
